# Cinnamaldehyde Attenuates Hyperuricemia-Associated Renal Injury by Modulating Urate Transporters and AIF1- and CMPK2/NLRP3-Related Inflammatory Signaling

**DOI:** 10.3390/ph19081261

**Published:** 2026-08-10

**Authors:** Yongxin Sun, Hao Tan, Jingyu Zhang, Zengyu Zhang, Shuang Huai, Manli Wang

**Affiliations:** College of Pharmacy, Beihua University, Jilin 132013, China

**Keywords:** cinnamaldehyde, hyperuricemia, NLRP3 inflammasome, CMPK2, AIF1

## Abstract

**Background:** Hyperuricemia is a well-established risk factor for both chronic kidney disease and gout. Currently available urate-lowering drugs, however, are frequently compromised by hepatorenal toxicity and gastrointestinal adverse effects. Cinnamaldehyde, the principal bioactive constituent of the traditional Chinese medicine cinnamon, has documented renoprotective properties, yet its integrated effects on hyperuricemia-associated renal injury remain incompletely defined. **Objectives:** This study investigated the therapeutic effects of cinnamaldehyde on hyperuricemia and explored the molecular mechanisms involved. **Materials and Methods:** An in vivo hyperuricemia model was established in KM mice by co-administration of potassium oxonate and hypoxanthine for 14 consecutive days, and an in vitro injury model was generated by exposing HK-2 human renal tubular epithelial cells to uric acid. For the in vivo study, mice were divided into five groups (control, model, febuxostat, low-dose CA, and high-dose CA; *n* = 9 per group) and were treated by oral gavage. Serum biochemical indices, renal and intestinal histopathology, inflammatory cytokines, renal urate transporter proteins (OAT1, OCT2, ABCG2, and SLC2A9), and components related to AIF1 and CMPK2/NLRP3 inflammasome signaling (ASC, caspase-1, IL-18, and IL-1β) were examined. Western blotting, qPCR, immunofluorescence, apoptosis analysis, mitochondrial membrane potential assessment, transmission electron microscopy, siRNA-mediated knockdown, and 16S rRNA gene sequencing were used to characterize the relevant mechanisms. **Results:** CA reduced serum uric acid (*p* ≤ 0.001 vs. model), creatinine (*p* ≤ 0.01 vs. model), and blood urea nitrogen (*p* ≤ 0.01 vs. model), alleviated renal and intestinal histopathological injury, and decreased circulating and renal inflammatory cytokines. Cinnamaldehyde increased ABCG2, OAT1, and OCT2 protein expression and reduced SLC2A9 expression (all *p* ≤ 0.05 vs. model). In vivo and in vitro, cinnamaldehyde suppressed AIF1- and CMPK2/NLRP3-related inflammatory proteins, reduced uric acid-induced apoptosis, and preserved mitochondrial membrane potential and ultrastructure. Fecal 16S rRNA sequencing suggested changes in selected microbial taxa, although alpha-diversity and BrayCurtis-based PERMANOVA/ANOSIM analyses did not demonstrate significant global community separation. **Conclusions:** These findings suggest that CA alleviates hyperuricemia-associated renal injury by regulating renal urate transporters, attenuating AIF1- and CMPK2/NLRP3-related inflammatory signaling, accompanied by compositional shifts in selected gut microbial taxa that warrant further mechanistic investigation. The study provides experimental support for further evaluation of cinnamaldehyde as a multi-target candidate for hyperuricemia-related renal injury.

## 1. Introduction

Hyperuricemia is a metabolic disorder characterized by persistently elevated serum uric acid, usually resulting from increased purine metabolism, excessive urate production, or insufficient urate excretion [1]. Its prevalence has continued to rise worldwide, and it is closely associated with gout, chronic kidney disease, and other metabolic complications [2]. In the kidney, long-term urate overload can promote tubular injury, inflammatory infiltration, and tubulointerstitial fibrosis, thereby contributing to the development of hyperuricemic nephropathy (HN) [3].

Uric acid homeostasis depends largely on coordinated renal and intestinal excretion. Approximately 70% of uric acid is eliminated by the kidney, whereas the remaining 30% is excreted through the intestine [4]. Several natural bioactive compounds have been reported to lower urate levels by regulating urate transporters. For example, punicalagin promotes urate elimination by upregulating ABCG2 and OAT1 in the kidney and intestine, whereas harpagoside inhibits urate reabsorption by downregulating URAT1 and GLUT9 (SLC2A9) [5,6]. These findings indicate that transporter regulation is a relevant strategy for improving urate excretion.

However, hyperuricemia is not only a disorder of urate accumulation. Persistently elevated uric acid can stimulate immune responses and sustain low-grade inflammation, which in turn aggravates renal injury and contributes to complications such as gout and cardiovascular disease [7]. Therefore, an effective intervention for hyperuricemia-associated renal injury may need to address both urate handling and inflammatory damage.

Among the inflammatory pathways implicated in urate-induced renal injury, AIF1 and CMPK2/NLRP3-related inflammatory signaling have recently attracted attention. AIF1 was selected in the present study because previous work in urate-stimulated human tubular epithelial cells reported that the lincRNA-p21/AIF1/CMPK2/NLRP3 pathway was associated with exosome-mediated inflammation, autophagy, and apoptosis [8]. This finding provides a disease-relevant basis for examining AIF1 in renal tubular injury, despite its more common use as an inflammatory marker in macrophages and μglia. AIF1 is a calcium-binding protein involved in inflammatory and fibrotic responses, including diabetic nephropathy and renal interstitial inflammationand [9,10,11]. CMPK2 localizes to mitochondria and has been linked to NLRP3 inflammasome activation through mitochondrial DNA-related signaling [12,13]. Activated NLRP3 promotes maturation of IL-1β and IL-18, thereby amplifying inflammatory injury [14,15,16]. In addition, hyperuricemia-associated gut dysbiosis may further aggravate renal inflammation through gut-derived metabolites and inflammasome activation [17]. These observations suggest a possible connection among urate transport, mitochondrial stress, inflammasome activation, and gut microbiota imbalance [18,19,20,21].

Cinnamaldehyde (CA), the principal bioactive aldehyde constituent of cinnamon bark, has demonstrated unequivocal renoprotective effects [22] and exerts broad-spectrum anti-inflammatory activity across multiple disease models by inhibiting the NLRP3 inflammasome and NF-κB signalling pathways [23,24]. Given that aberrant NLRP3 inflammasome activation is an established pathogenic mechanism in hyperuricemia [25], we sought to determine whether CA can attenuate hyperuricemia-associated renal injury.

Although CA has been reported to lower serum urate by inhibiting xanthine oxidase and modulating urate transporters [26], and to exert anti-inflammatory effects through NLRP3 inflammasome suppression in other disease contexts [24,27], its effects on hyperuricemia-associated renal tubular injury remain incompletely understood. In particular, it is unclear whether the renoprotective effects of cinnamaldehyde are associated with AIF1- and CMPK2/NLRP3-related inflammatory signaling and how alterations in urate transporter expression, mitochondrial homeostasis, and inflammatory responses are related within the same experimental setting. Moreover, the potential association between cinnamaldehyde treatment and gut microbial composition during hyperuricemia has not been adequately characterized. Therefore, using complementary mouse and uric acid-exposed HK-2 cell models, the present study systematically evaluated the effects of cinnamaldehyde on renal function, urate transporter expression, mitochondrial injury, AIF1- and CMPK2/NLRP3-related inflammatory signaling, and fecal microbial composition. The novelty of this study lies in the integrated assessment of these molecular and physiological changes, together with exploratory analysis of microbiota alterations, rather than in proposing the gut microbiota as an established causal mechanism.

## 2. Results

### 2.1. Cinnamaldehyde Attenuates Hyperuricemia and Renal Injury

**Figure 1:**
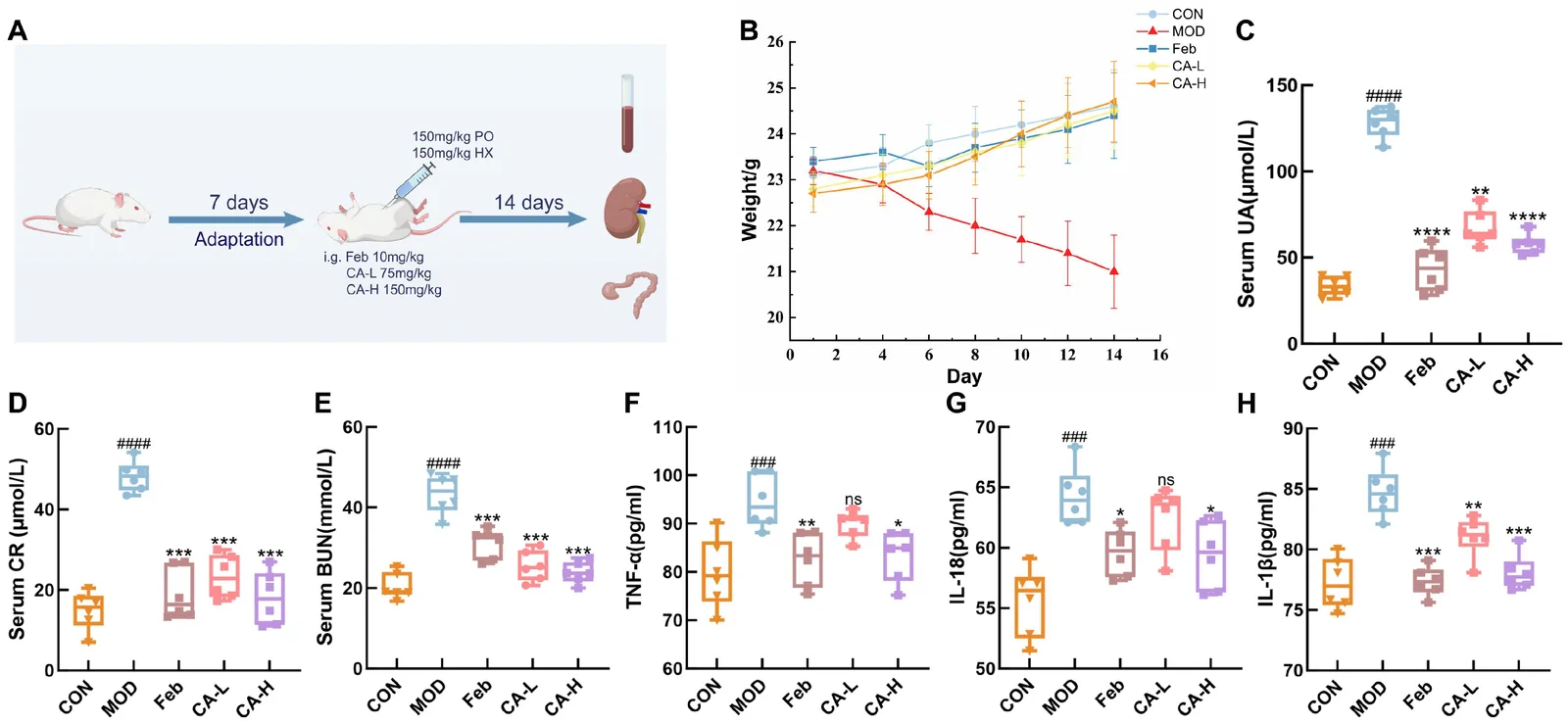
Effects of cinnamaldehyde on renal injury markers in hyperuricemic mice. (**A**) Schematic diagram of the experimental protocol. Hyperuricemia was induced in all groups except the control group by intraperitoneal injection of potassium oxonate and hypoxanthine. Two hours later, treatment groups received febuxostat or cinnamaldehyde by oral gavage, whereas the control and model groups received vehicle; this regimen was maintained daily for 14 consecutive days. (**B**) Weight change. (**C**) Serum uric acid levels. (**D**) Serum creatinine levels. (**E**) Serum urea nitrogen levels. (**F**–**H**) Serum levels of TNF-α, IL-1β, and IL-18. Data are presented as mean ± SD (*n* = 6 per group). Statistical analysis was performed by one-way ANOVA followed by Tukey’s multiple-comparison test. * *p* ≤ 0.05, ** *p* ≤ 0.01, *** *p* ≤ 0.001, and **** *p* ≤ 0.0001 vs. model; ### *p* ≤ 0.001, and #### *p* ≤ 0.0001 vs. control. ns indicates not significant (*p* ≥ 0.05). Shapes and colors represent different groups.

We first established a hyperuricemic mouse model using potassium oxonate plus hypoxanthine to assess the therapeutic effect of cinnamaldehyde (Figure 1A). As shown in Figure 1B, the control group mice exhibited a steady increase in body weight, while the model group mice showed a gradual decrease. The Feb and CA treatment groups demonstrated a steady increase in body weight. After 14 days of induction, the model group showed increased serum uric acid, creatinine, and blood urea nitrogen compared with the control group, indicating impaired renal excretory function (Figure 1C–E). In contrast, cinnamaldehyde administration (75 and 150 mg/kg) reduced all three parameters in a dose-dependent manner, and the high-dose effect was close to that of febuxostat (10 mg/kg). Serum IL-1β, IL-18, and TNF-α were also increased in the model group and were reduced by cinnamaldehyde treatment, particularly at the high dose (Figure 1F–H).

### 2.2. Cinnamaldehyde Attenuates Renal and Intestinal Histopathological Injury

**Figure 2:**
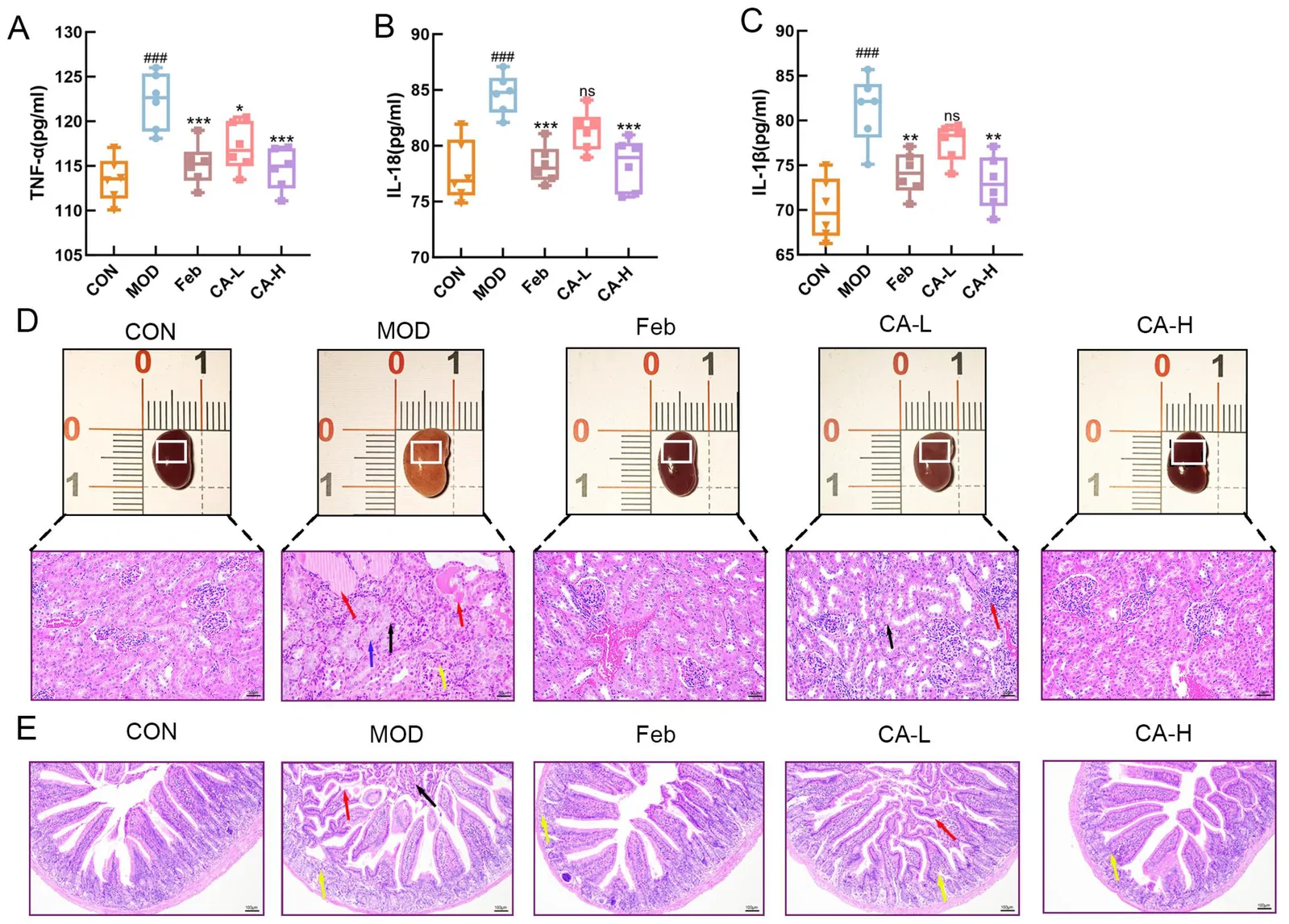
Cinnamaldehyde ameliorates renal and intestinal pathological alterations in hyperuricemic mice. (**A**–**C**) Renal levels of the pro-inflammatory cytokines TNF-α, IL-18, and IL-1β. (**D**) Representative hematoxylin and eosin (H&E) staining of kidney sections. (**E**) Representative H&E staining of intestinal sections. Scale bar, 50 μm; original magnification, 200×. Red arrows indicate renal tubules; black arrows denote inflammatory cell infiltration. Data are presented as mean ± SD (*n* = 6 per group). Statistical analysis for cytokine data was performed by one-way ANOVA followed by Tukey’s multiple-comparison test. * *p* ≤ 0.05, ** *p* ≤ 0.01, *** *p* ≤ 0.001 vs. model; ### *p* ≤ 0.001 vs. control. ns indicates not significant (*p* ≥ 0.05). Shapes and colors represent different groups.

As shown in Figure 2A–C, renal IL-18, IL-1β, and TNF-α levels were increased in the model group compared with the control group and were reduced by febuxostat and high-dose cinnamaldehyde. Low-dose cinnamaldehyde showed a weaker effect on IL-18 and IL-1β. H&E staining showed normal renal architecture in the control group, with no obvious inflammatory cell infiltration. In the model group, renal tubular epithelial degeneration (black arrows), interstitial inflammatory infiltration (red arrows), local fibroplasia (blue arrows), and poorly defined glomerular boundaries were observed (yellow arrows). Cinnamaldehyde treatment alleviated these pathological changes (Figure 2D). Intestinal sections from the control group showed preserved mucosal structure, whereas the model group showed disorganized villi, focal epithelial erosion and shedding (red arrows), sloughed tissue debris in the intestinal lumen (black arrows), and inflammatory cell infiltration in the mucosa (yellow arrows). Cinnamaldehyde treatment partially restored mucosal integrity (Figure 2E).

### 2.3. Cinnamaldehyde Restores Renal Urate Transporter Expression and Suppresses the AIF1- and CMPK2/NLRP3-Related Inflammatory Signaling

**Figure 3:**
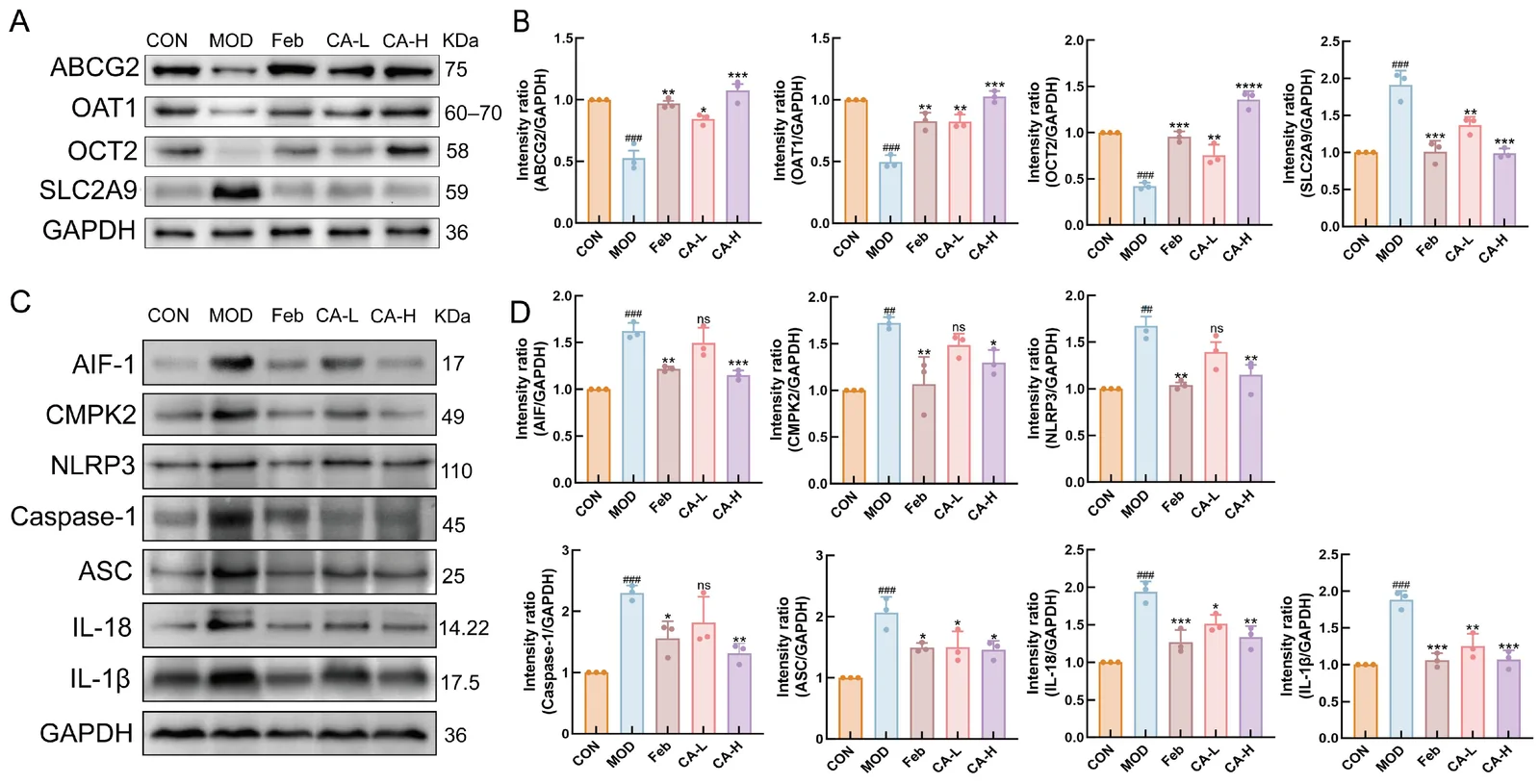
Cinnamaldehyde (CA) normalizes aberrant expression of renal urate transporters and suppresses inflammatory responses in hyperuricemic mice. (**A**) Representative Western blot images of ABCG2, OAT1, OCT2, and SLC2A9. (**B**) Densitometric quantification of ABCG2, OAT1, OCT2, and SLC2A9 protein levels. (**C**) Representative Western blot images of AIF-1, CMPK2, NLRP3, Caspase-1, ASC, IL-18, and IL-1β. (**D**) Densitometric quantification of AIF-1, CMPK2, NLRP3, Caspase-1, ASC, IL-18, and IL-1β protein levels. Data are expressed as mean ± SEM (*n* = 6 per group), and all Western blot analyses were performed in triplicate. Statistical analysis was performed by one-way ANOVA followed by Tukey’s multiple-comparison test. * *p* ≤ 0.05, ** *p* ≤ 0.01, *** *p* ≤ 0.001, **** *p* ≤ 0.0001 vs. model; ## *p* ≤ 0.01, ### *p* ≤ 0.001, vs. control. “ns” indicates not significant (*p* ≥ 0.05). Colors represent different groups.

Western blot analysis of renal urate transporters showed that ABCG2, OCT2, and OAT1 protein levels were decreased in the model group, whereas SLC2A9 expression was increased compared with the control group. Cinnamaldehyde treatment reversed these abnormal expression patterns, increasing ABCG2, OAT1, and OCT2 and decreasing SLC2A9 (Figure 3A,B).

Because impaired renal urate excretion is closely associated with inflammation, we next examined whether cinnamaldehyde affected the AIF1- and CMPK2/NLRP3-related inflammatory signaling. Compared with the control group, the model group showed increased renal protein levels of AIF1, CMPK2, NLRP3, ASC, caspase-1, mature IL-18, and mature IL-1β. Cinnamaldehyde treatment, especially at the high dose, reduced the expression of these proteins (Figure 3C,D). These results indicate that cinnamaldehyde regulates urate transporter expression and attenuates renal inflammatory activation associated with the AIF1- and CMPK2/NLRP3-related inflammatory signaling.

### 2.4. Cinnamaldehyde Is Associated with Changes in Selected Gut Microbial Taxa in Hyperuricemic Mice

**Figure 4:**
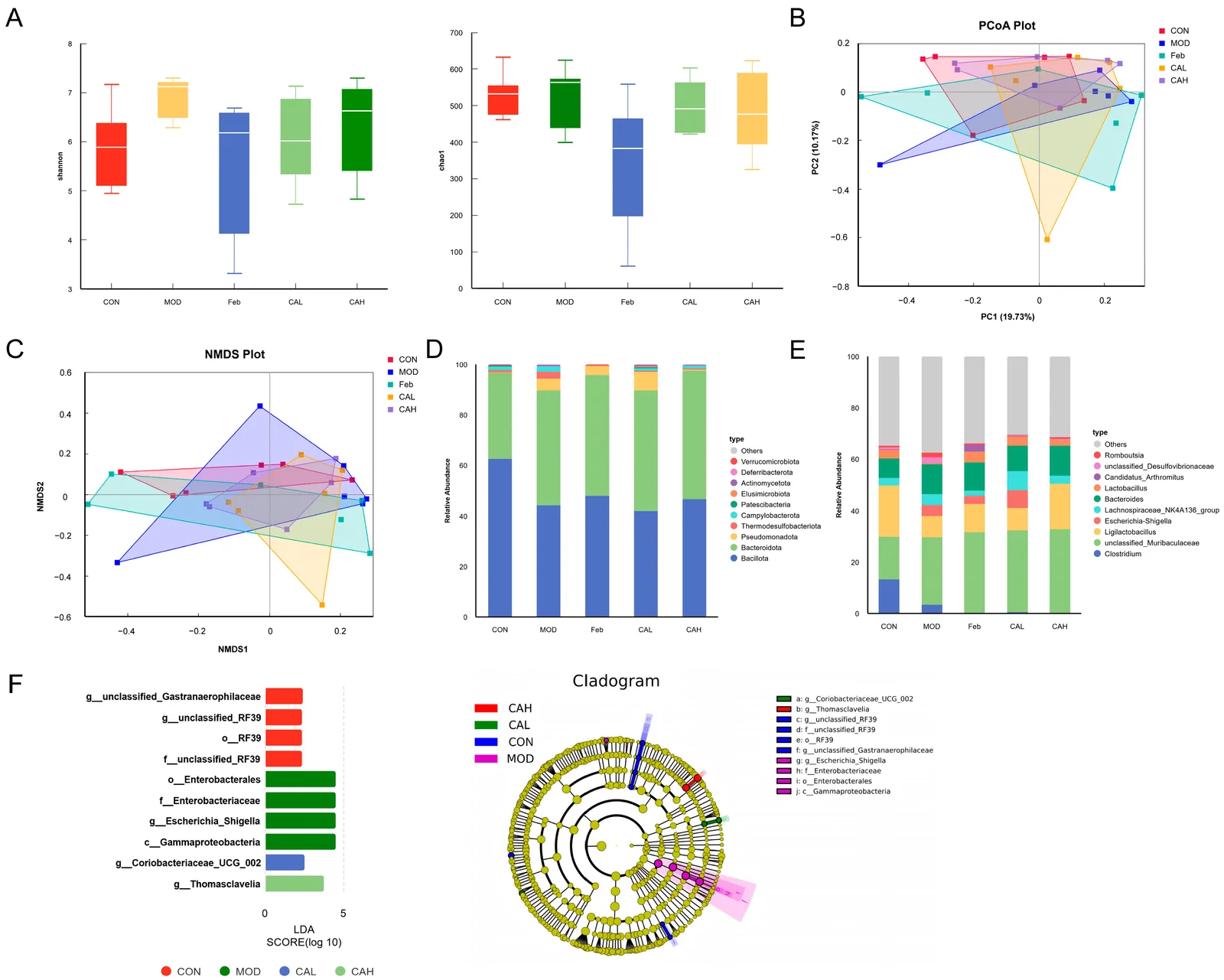
Gut microbiota analysis based on 16S rRNA gene sequencing in hyperuricemic mice. (**A**) Shannon alpha-diversity index. (**B**) Principal coordinate analysis (PCoA) based on BrayCurtis distances. (**C**) Non-metric multidimensional scaling (NMDS) based on BrayCurtis distances. Pairwise PERMANOVA and ANOSIM analyses did not identify significant overall community separation between the model and treatment groups. (**D**) Phylum-level community composition. (**E**) Relative abundance of selected genera. (**F**) Cladogram illustrating bacteria with significant differences among groups according to linear discriminant analysis (LDA) effect size (LEfSe) analysis, with LDA scores > 2.5 and adjusted *p* < 0.05. Data are shown for fecal 16S rRNA sequencing samples (*n* = 6 per group). Alpha-diversity indices were compared using non-parametric rank-based tests; beta diversity was evaluated using PERMANOVA and ANOSIM based on BrayCurtis distances; taxa-level differences were explored using Wilcoxon or KruskalWallis tests and LEfSe analysis as appropriate. Taxa-level results should be interpreted as exploratory compositional findings.

Because the intestine is an important route for uric acid excretion, we next examined the gut microbiota using 16S rRNA gene amplicon sequencing of fecal samples (*n* = 6 per group). The sequencing depth was sufficient for community profiling, with Good’s coverage values approaching 1.000 in all groups. Alpha-diversity indices showed no statistically significant pairwise differences among groups for Shannon, Chao1, or observed features (Figure 4A). BrayCurtis PCoA and NMDS plots suggested treatment-related clustering tendencies, but pairwise PERMANOVA and ANOSIM did not detect significant overall community separation (for example, Control vs. MOD: PERMANOVA *p* = 0.267, ANOSIM *p* = 0.326; MOD vs. CA.H: PERMANOVA *p* = 0.550, ANOSIM *p* = 0.404; Figure 4B,C and Table S1). Therefore, the microbiota results were interpreted mainly at the level of compositional shifts rather than as evidence of a robust global community restructuring. At the phylum level, the model group showed a lower *Bacillota*/*Bacteroidota* ratio than controls (0.97 vs. 1.84), while the high-dose cinnamaldehyde group remained close to the model pattern (0.91), indicating that this ratio was not clearly restored by cinnamaldehyde (Figure 4D). At the genus level, *Escherichia*-*Shigella* was nearly absent in controls (0.001%) but increased in the model group (4.30%); this genus decreased to 0.004% after high-dose cinnamaldehyde treatment, although the MOD vs. CA.H pairwise Wilcoxon comparison did not remain significant after correction (Figure 4E and Table S1). LEfSe analysis identified several discriminative taxa across groups, including enrichment of *Enterobacterales*/*Enterobacteriaceae*/*Escherichia*-*Shigella*-related taxa in the model group (Figure 4F). Overall, cinnamaldehyde treatment was associated with changes in selected microbial taxa, whereas global microbiota diversity and beta-diversity differences were modest.

### 2.5. Cinnamaldehyde Exerts Anti-Inflammatory and Anti-Apoptotic Effects in Uric Acid-Treated HK-2 Cells

**Figure 5:**
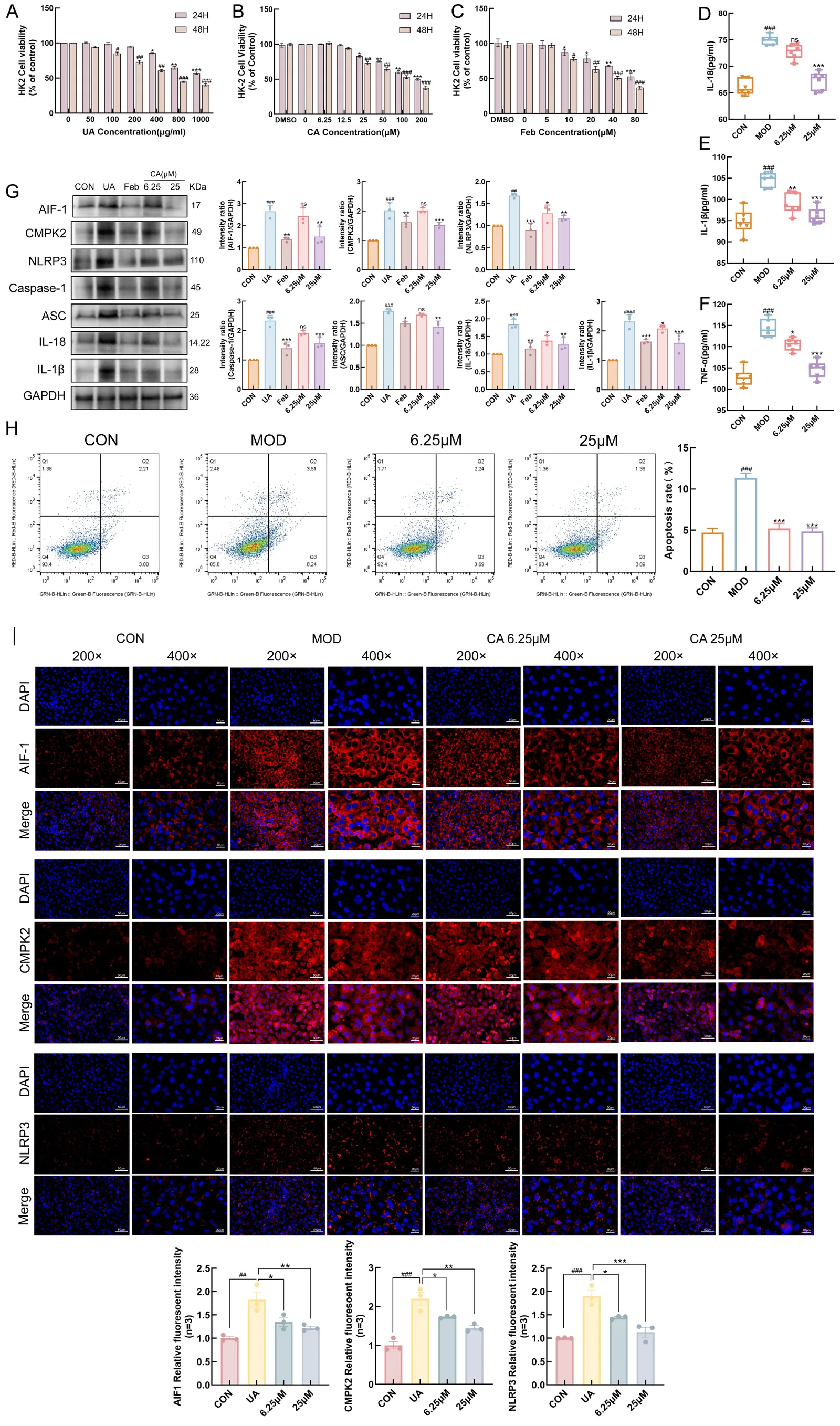
Cinnamaldehyde ameliorates uric acid-induced injury and inflammatory response in HK-2 cells. (**A**) Cell viability of HK-2 cells exposed to increasing concentrations of uric acid (0–1000 μg/mL) for 24 and 48 h, as determined by CCK-8 assay. (**B**,**C**) Viability of HK-2 cells treated with cinnamaldehyde (0–200 μM) or febuxostat (0–80 μM) for 24 and 48 h. (**D**–**F**) Levels of IL-18, IL-1β, and TNF-α released into the culture supernatant. (**G**) Representative Western blot bands and densitometric quantification of AIF-1, CMPK2, NLRP3, Caspase-1, ASC, IL-18, and IL-1β protein levels (*n* = 3). (**H**) Apoptosis of HK-2 cells. (**I**) Representative immunofluorescence images of AIF-1, CMPK2, and NLRP3 (*n* = 3; scale bars/magnification are indicated in the images). Western blot data are presented as mean ± SEM from three independent experiments; unless otherwise specified, data are presented as mean ± SD. Statistical analysis was performed by one-way ANOVA followed by Tukey’s multiple-comparison test. * *p* ≤ 0.05, ** *p* ≤ 0.01, *** *p* ≤ 0.001 vs. model; # *p* ≤ 0.05, ## *p* ≤ 0.01, ### *p* ≤ 0.001, #### *p* ≤ 0.0001 vs. control. ns indicates not significant (*p* ≥ 0.05). Shapes and colors represent different groups.

To further examine the underlying mechanisms, we used a uric acid-induced injury model in HK-2 cells. The working concentrations of uric acid (400 μg/mL), cinnamaldehyde (6.25 and 25 μM), and febuxostat (10 μM) were selected based on cell viability assays (Figure 5A–C). Uric acid stimulation increased the secretion of IL-18, IL-1β, and TNF-α, and this response was reduced by cinnamaldehyde co-treatment. Immunoblotting showed a similar pattern: uric acid increased the expression of NLRP3, ASC, caspase-1, and the mature forms of IL-1β and IL-18, whereas cinnamaldehyde co-treatment reduced these changes (Figure 5D–G). Flow cytometry showed that uric acid increased the proportion of apoptotic cells, and this increase was attenuated by both low- and high-dose cinnamaldehyde (Figure 5H). Immunofluorescence imaging was consistent with these results (Figure 5I).

### 2.6. Cinnamaldehyde Attenuates Uric Acid-Induced Mitochondrial Damage in HK-2 Cells

**Figure 6:**
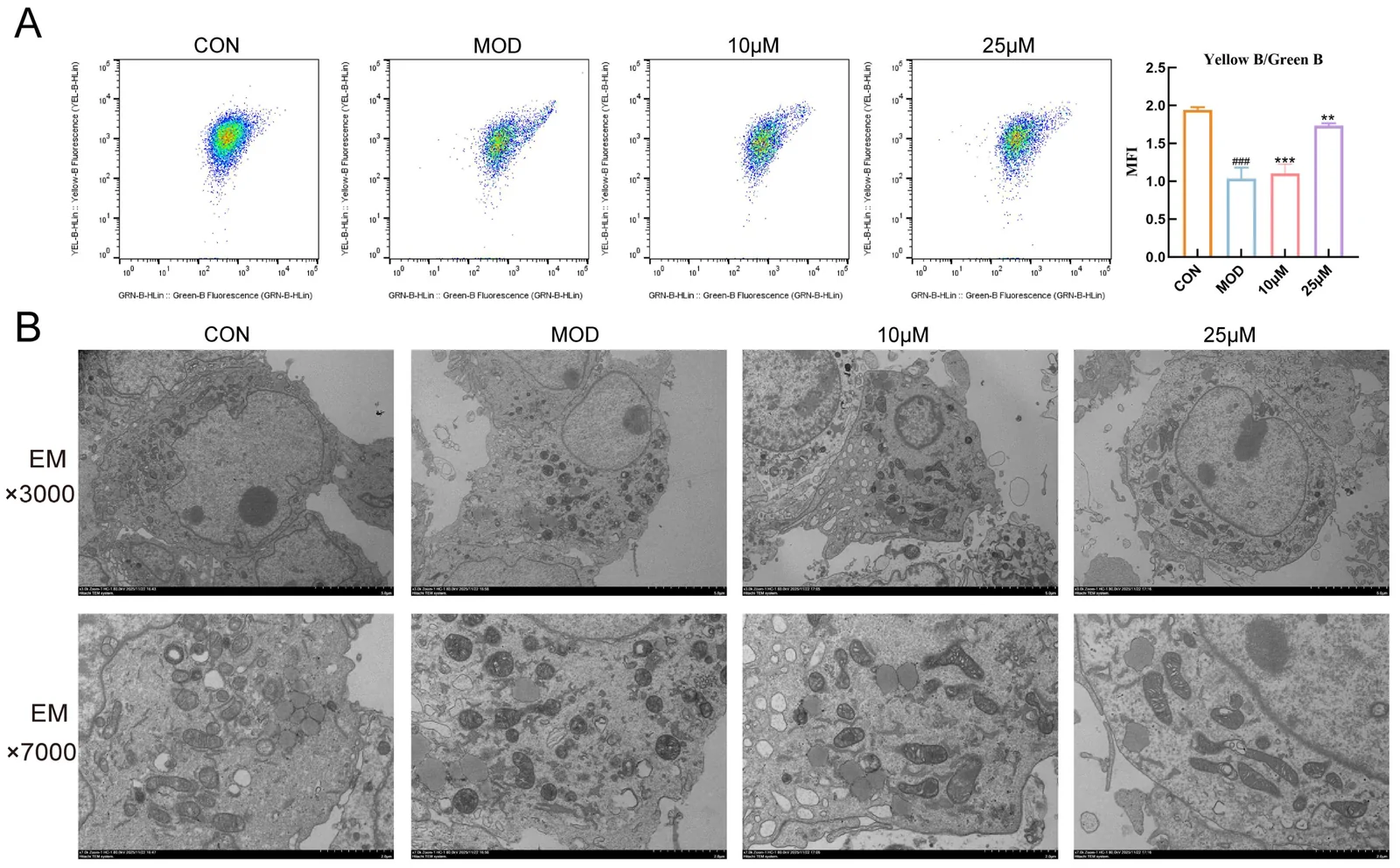
Cinnamaldehyde attenuates uric acid-induced mitochondrial damage in HK-2 cells. (**A**) Changes in mitochondrial membrane potential (MMP). (**B**) Alterations in mitochondrial ultrastructure. Data are expressed as mean ± SD (*n* = 3 per group). Statistical analysis was performed by one-way ANOVA followed by Tukey’s multiple-comparison test. ** *p* ≤ 0.01, *** *p* ≤ 0.001 vs. model; ### *p* ≤ 0.001 vs. control. Shapes and colors represent different groups.

Given the close relationship between mitochondrial integrity and NLRP3 inflammasome activation, and the mitochondrial localization of CMPK2, we next assessed mitochondrial membrane potential (ΔΨm) and ultrastructure. Uric acid treatment reduced ΔΨm in HK-2 cells and, in electron micrographs, caused mitochondrial swelling, electron-lucent matrices, and cristae disruption. Co-treatment with cinnamaldehyde preserved ΔΨm and improved mitochondrial morphology, with more elongated mitochondria and better-organized cristae (Figure 6A,B). These observations suggest that cinnamaldehyde-mediated regulation of the AIF1- and CMPK2/NLRP3-related inflammatory signaling is associated with preservation of mitochondrial homeostasis.

### 2.7. Cinnamaldehyde Attenuates Uric Acid-Induced Injury in HK-2 Cells via the AIF1- and CMPK2/NLRP3-Related Inflammatory Signaling Pathway

**Figure 7:**
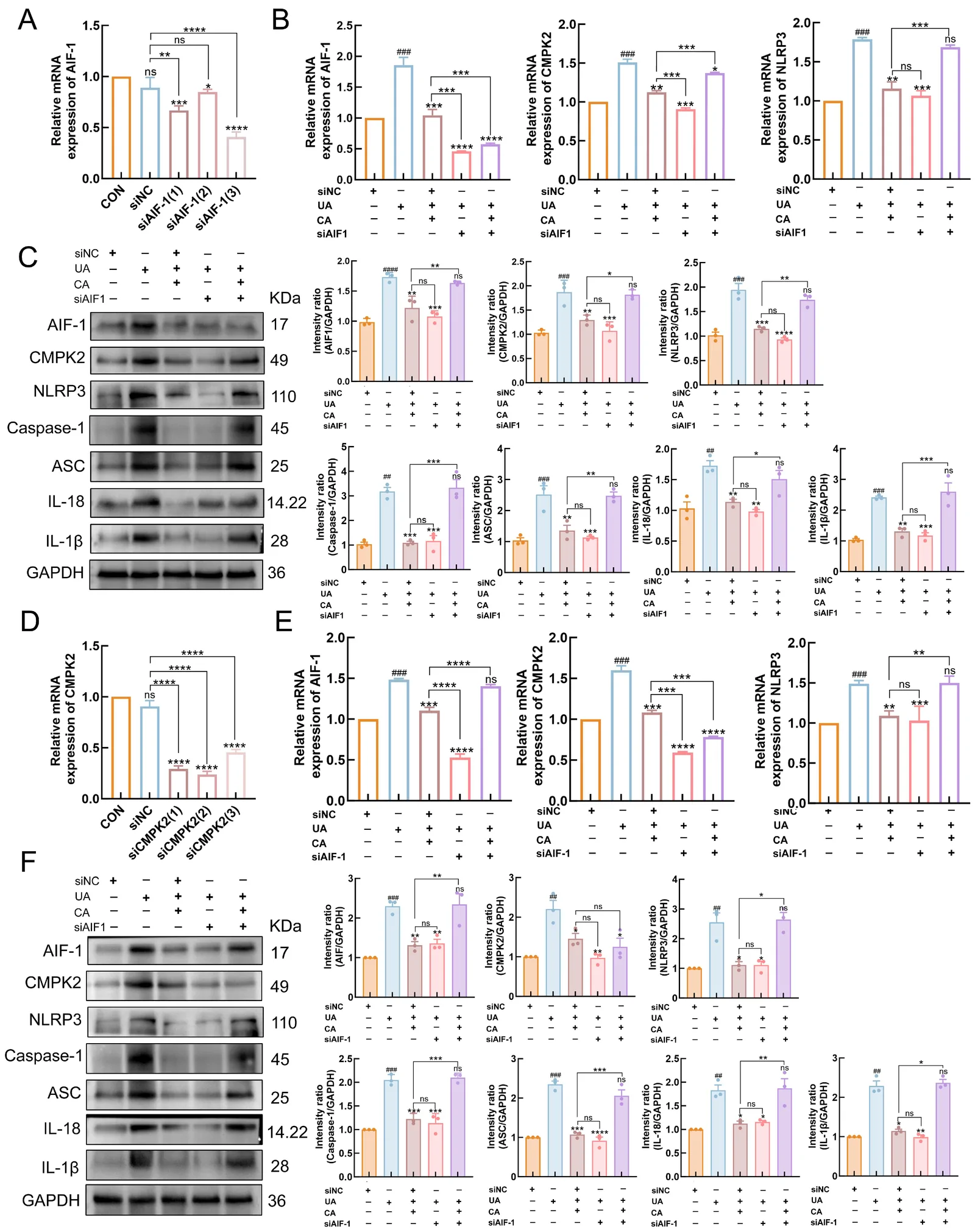
Cinnamaldehyde modulates the AIF-1 and CMPK2/NLRP3-related signaling. (**A**) Aif-1 transcript levels following siRNA-mediated knockdown of Aif-1 in HK-2 cells (*n* = 3). (**B**) Transcript levels of Aif-1, Cmpk2, and Nlrp3 in the indicated groups of siAIF-1-transfected HK-2 cells (*n* = 3). (**C**) Representative immunoblot bands and densitometric quantification of AIF-1, CMPK2, NLRP3, Caspase-1, ASC, IL-18, and IL-1β in siAIF-1-transfected HK-2 cells (*n* = 3). (**D**) Cmpk2 transcript levels after siRNA-mediated silencing of Cmpk2 (*n* = 3). (**E**) Transcript levels of Aif-1, Cmpk2, and Nlrp3 in the indicated groups of siCMPK2-transfected HK-2 cells (*n* = 3). (**F**) Representative immunoblot bands and corresponding quantification of AIF-1, CMPK2, NLRP3, Caspase-1, ASC, IL-18, and IL-1β in siCMPK2-transfected HK-2 cells (*n* = 3). Data are presented as mean ± SEM. Statistical analysis was performed by one-way ANOVA followed by Tukey’s multiple-comparison test. * *p* ≤ 0.05, ** *p* ≤ 0.01, *** *p* ≤ 0.001, **** *p* ≤ 0.0001 vs. model; ## *p* ≤ 0.01, ### *p* ≤ 0.001, #### *p* ≤ 0.0001 vs. control. ns indicates not significant (*p* ≥ 0.05). Colors represent different groups.

To test whether the AIF1- and CMPK2/NLRP3-related inflammatory signaling contributes to the protective effect of cinnamaldehyde, we silenced AIF1 or CMPK2 in HK-2 cells using specific siRNAs. Knockdown was confirmed by reduced AIF1 and CMPK2 mRNA levels relative to control cells (Figure 7A,D). After AIF1 silencing, the inhibitory effect of cinnamaldehyde on CMPK2 and NLRP3 protein expression was weakened (Figure 7B,C). Similarly, after CMPK2 knockdown, cinnamaldehyde no longer clearly reduced NLRP3 and its downstream effectors, while AIF1 levels were largely unchanged (Figure 7E,F). These data support the involvement of the AIF1- and CMPK2/NLRP3-related cascade in the protective action of cinnamaldehyde.

## 3. Discussion

In this study, cinnamaldehyde alleviated hyperuricemia-associated renal injury in a potassium oxonate/hypoxanthine-induced mouse model and reduced uric acid-induced injury in HK-2 cells. The protective pattern was reflected by decreased serum uric acid and renal injury markers, improved renal and intestinal histopathology, normalization of renal urate transporter expression, reduced inflammatory cytokine production, preservation of mitochondrial morphology and membrane potential, and changes in selected gut microbial taxa. Unlike previous studies that have focused narrowly on isolated mechanisms—such as XOD inhibition or NLRP3 suppression [28,29]—these findings reveal that cinnamaldehyde operates through an integrated, multi-nodal network that coordinates urate handling, inflammatory signalling, mitochondrial homeostasis, and gut microbial equilibrium.

Regulation of urate transporters appears to be an important component of this protective profile [30]. Hyperuricemia is sustained not only by increased urate generation but also by impaired renal and intestinal excretion [31,32]. Han et al. [6] showed that punicalagin reduces serum uric acid by upregulating ABCG2 and OAT1 in both the kidney and intestine, whereas Fu et al. [5] reported that harpagide achieves a similar urate-lowering effect through suppression of URAT1 and GLUT9. In the present model, hyperuricemia was accompanied by decreased expression of the secretory transporters OAT1, OCT2, and ABCG2 and increased expression of the reabsorptive transporter SLC2A9. Cinnamaldehyde reversed these changes, producing a transporter profile consistent with enhanced urate elimination. Similar transporter-oriented mechanisms have been reported for several natural products, including Benincasae Exocarpium, Polygonati Rhizoma polysaccharide, and polysaccharide-protein complexes [33,34,35,36]. These observations support the view that transporter modulation is one plausible route by which cinnamaldehyde lowers serum urate. Nevertheless, protein expression does not necessarily equal transporter activity, and functional urate uptake or efflux assays would be needed to confirm the transport capacity directly. For OAT1 and OCT2, which mediate basolateral uptake of urate and organic anions/cations, respectively, their net contribution to urate secretion depends not only on abundance but also on membrane localization, substrate availability, and transmembrane gradients. Likewise, ABCG2-mediated apical efflux and SLC2A9-mediated reabsorption are subject to post-translational regulation and may not correlate linearly with protein expression [35]. Therefore, direct functional assays, such as transporter-specific urate uptake or efflux measurements are required to confirm whether the observed expression changes result in altered net urate transport capacity. Accordingly, the present findings demonstrate that cinnamaldehyde reverses model-associated alterations in transporter protein expression but do not establish direct enhancement or inhibition of transporter activity.

Inflammatory injury provides a second layer of explanation for the renoprotective effect of cinnamaldehyde. Sustained urate accumulation can promote renal inflammation and accelerate tissue injury [37]. Cinnamaldehyde has been independently shown to suppress NLRP3 inflammasome activation in models of rheumatoid arthritis [28] and gonococcal infection [27]. However, neither study explored its potential involvement—nor the roles of AIF1 and CMPK2—in the context of hyperuricaemia-induced tubular injury, a critical yet underinvestigated pathway in gouty nephropathy. In this study, hyperuricemic mice and uric acid-stimulated HK-2 cells showed increased AIF1, CMPK2, NLRP3, ASC, caspase-1, mature IL-1β, and IL-18, whereas cinnamaldehyde reduced these inflammatory signals. The consistency between the animal and cell models strengthens the interpretation that cinnamaldehyde does not merely lower serum urate but also attenuates urate-associated inflammatory activation. This is relevant because inflammation can further impair tubular function, amplify cytokine production, and aggravate renal structural damage even when urate levels begin to decline.

AIF1 and CMPK2/NLRP3-related signaling may both participate in urate-induced inflammatory activation, but the present data should not be interpreted as proving a direct linear AIF1-to-CMPK2 signaling pathway. The rationale for examining this pathway is supported by previous work showing that lincRNA-p21/AIF1/CMPK2/NLRP3 signaling promoted inflammation, autophagy, and apoptosis in urate-stimulated human tubular epithelial cells through exosome-related mechanisms [8]. Thus, AIF1 and CMPK2/NLRP3 represent a disease-relevant candidate pathway in tubular epithelial injury rather than an arbitrary target set. Nevertheless, AIF1 is commonly studied as a calcium-binding protein and inflammatory marker in macrophages and microglia [38], whereas CMPK2 is a mitochondrial nucleotide kinase [39], and the molecular mechanism linking AIF1 to CMPK2 remains insufficiently defined. In the present study, uric acid-induced injury was associated with increased AIF1 and CMPK2 expression, and siRNA-mediated knockdown of AIF1 or CMPK2 weakened the regulatory effect of cinnamaldehyde on downstream NLRP3-related proteins. These findings support an association among AIF1, CMPK2, and NLRP3 activation in this model. They do not establish direct signal transmission from AIF1 to CMPK2, and the observed changes may also reflect indirect or parallel inflammatory responses.

Mitochondrial protection is a mechanistically important finding because mitochondrial dysfunction is a recognized upstream trigger of NLRP3 inflammasome activation [40]. CMPK2 is localized to mitochondria and has been linked to mitochondrial DNA-dependent inflammatory signaling, including mtDNA-STING-related NLRP3 activation, in several disease contexts [12,13]. In the present study, uric acid exposure caused loss of mitochondrial membrane potential, mitochondrial swelling, electron-lucent matrices, and cristae disruption, whereas cinnamaldehyde preserved ΔΨm and improved mitochondrial ultrastructure. These observations suggest that cinnamaldehyde may reduce mitochondrial stress signals that favor NLRP3 activation. However, mtDNA release, STING activation, and related upstream events were not directly measured, so this mechanism remains inferential.

The gut microbiota findings add another layer to the multi-system nature of hyperuricemia, but they should be interpreted with care. The intestine is an important route for uric acid excretion, and gut microbial metabolism can influence host purine and urate homeostasis [41,42]. In the present dataset, alpha-diversity indices were not significantly different among groups, and BrayCurtis PERMANOVA/ANOSIM did not support statistically significant pairwise separation of overall microbial community structure. Nevertheless, the taxonomic profiles showed biologically relevant compositional changes, including a marked model-associated increase in *Escherichia*-*Shigella* that was lower in the high-dose cinnamaldehyde group. These findings are consistent with previous reports linking hyperuricemia, intestinal dysbiosis, and renal inflammation [17,23,43], but they should be presented as exploratory microbiota associations rather than proof of microbiota-driven causality. Without targeted metabolomics, fecal microbiota transplantation, antibiotic-depletion experiments, or direct measurements of intestinal urate transport, the gut microbiota results support a possible gut-kidney association but do not establish a causal microbiota-metabolite-kidney axis.

It is noteworthy that cinnamaldehyde, as a pleiotropic bioactive compound, exerts pharmacological effects beyond urate transport and inflammation. Previous studies have shown that cinnamaldehyde can ameliorate insulin resistance [44]. Insulin resistance and hyperuricemia are closely and potentially bidirectionally associated, and their coexistence may contribute to metabolic dysfunction and renal injury [44]. Therefore, improvement in insulin sensitivity may represent an additional mechanism through which cinnamaldehyde exerts reno-protective effects. However, fasting glucose, circulating insulin, and insulin sensitivity were not evaluated in the present short-term hyperuricemia model; consequently, the contribution of this mechanism cannot be determined from the current data. Future studies incorporating metabolic phenotyping are needed to clarify this possibility.

Taken together, the results suggest a working model in which cinnamaldehyde reduces hyperuricemia-associated renal injury through several mutually reinforcing processes. Improved transporter expression may favor urate elimination; reduced AIF1- and CMPK2/NLRP3-related signaling may limit inflammatory amplification; preservation of mitochondrial structure may decrease inflammasome-triggering stress signals; and changes in selected gut microbial taxa may accompany improvement of the hyperuricemic state. This integrated interpretation fits the pharmacological characteristics of natural products, which often show moderate effects across multiple biological nodes rather than strong selectivity for a single target. Importantly, the current findings do not require the assumption that all observed effects are primary actions of cinnamaldehyde; some may be secondary to reduced urate burden or improved tissue injury.

A strength of the present study is the use of complementary in vivo and in vitro models, integrating biochemical, histopathological, inflammatory, mitochondrial, and urate transporter-related assessments with siRNA-mediated mechanistic perturbation and exploratory microbiota profiling. The generally consistent findings across the mouse and cell models support the biological relevance of the observed renoprotective effects. Nevertheless, the breadth of this experimental design does not establish a complete causal mechanism of cinnamaldehyde action. Several limitations should be considered when interpreting these findings. First, animal experiments were performed only in male mice, so possible sex-related differences in efficacy, metabolism, and safety remain unresolved; this is particularly relevant because hyperuricemia shows sex-related epidemiological differences. Second, the in vitro work relied on HK-2 cells, and validation in primary renal tubular epithelial cells would improve pathological relevance. In addition, AIF1 is widely recognized as a macrophage/microglial marker, and the present study did not determine whether renal AIF1 signals in vivo originated mainly from tubular epithelial cells or infiltrating immune cells. Third, transporter protein expression was measured, but functional urate transport was not directly tested. Fourth, the microbiota analysis was based on 16S rRNA sequencing and did not include metabolomic or causal validation, intestinal urate transporter measurements, or fecal microbiota transplantation. Fifth, ALT, AST, and other hepatic safety indices were not evaluated, which limits assessment of systemic safety. Sixth, daily food intake was not quantitatively recorded using metabolic cages. Although body weight trajectories are provided in Figure 1B, these data cannot exclude potential differences in food intake among groups. Future studies should include direct monitoring of food intake and related metabolic parameters.

## 4. Materials and Methods

### 4.1. Materials and Reagents

Potassium oxonate (PO, purity ≥ 98%, Cat. No. P137112), hypoxanthine (HX, purity ≥ 98%, Cat. No. H301755), and febuxostat (Feb, purity ≥ 98%, CAS No. 144060-53-7) were purchased from Aladdin Biochemical Technology Co., Ltd. (Shanghai, China). Cinnamaldehyde (CA, purity ≥ 98%, Cat. No. C822622) and uric acid (UA, purity ≥ 99%, CAS No. 69-93-2) were obtained from Macklin Biochemical Technology Co., Ltd. (Shanghai, China). Primary and secondary antibodies for western blotting were sourced as follows: OCT2 (A39260), ABCG2 (A78095), SLC2A9 (A54290), AIF-1 (A47071), NLRP3 (A49570), ASC (A15093), IL-18 (A60276), IL-1β (A13948), GAPDH (RA0003), goat anti-mouse IgG (H+L)-HRP (M00001), and goat anti-rabbit IgG (H+L)-HRP (R00001) were from Nature Biosciences (Hangzhou, China); OAT1 (DF6633) was from Affinity (Liyang, China); Caspase-1 (A0964) from Abclonal (Wuhan, China); and CMPK2 (PJP01659S) from Abmart (Shanghai, China).

### 4.2. Hyperuricemia Model Induction and Drug Administration

Male KM mice were obtained from Liaoning Changsheng Biotechnology Co., Ltd. (Benxi, China) and maintained under standard conditions. All animal experiments were conducted in compliance with the Guide for the Care and Use of Laboratory Animals of Beihua University and were approved by the Institutional Animal Ethics Committee of Beihua University (Approval No. 2025070803, approved on 8 July 2025).

After 7 days of acclimatization, mice were randomly divided into five groups by body weight (control, model, febuxostat (10 mg/kg), low-dose cinnamaldehyde (75 mg/kg), and high-dose cinnamaldehyde (150 mg/kg)), with 9 animals initially enrolled per group to account for a potential 10–15% attrition rate due to mortality or modeling failure [45]. Animals that completed the treatment protocol and yielded samples of sufficient quality were included in endpoint analyses, and exact sample sizes are indicated in the corresponding figure legends. From the first day of modeling, the control group received a daily intraperitoneal injection of an equal volume of saline, whereas the remaining four groups were injected intraperitoneally with a suspension containing potassium oxonate (150 mg/kg) and hypoxanthine (150 mg/kg) for 14 consecutive days [27]. Two hours after each daily injection, the corresponding intragastric interventions were administered: febuxostat (10 mg/kg), cinnamaldehyde at 75 or 150 mg/kg, or an equivalent volume of vehicle (0.9% saline + 0.5% sodium carboxymethyl cellulose). On day 14, mice were sacrificed 2 h after the final gavage, mice were deeply anesthetized with an intraperitoneal injection of sodium pentobarbital (50 mg/kg). After confirmation of the loss of the toe-pinch reflex, all mice were euthanized by cervical dislocation, and blood, kidney, ileal tissue, and fecal samples were collected immediately.

### 4.3. Haematoxylin and Eosin (H&E) Staining

Mouse kidney and ileal tissues were washed with PBS, fixed in 4% paraformaldehyde, embedded in paraffin, and sectioned. Sections were baked at 65 °C for 6 h and then stained with hematoxylin and eosin (H&E). Images were captured using a Nikon TS2 inverted microscope (Nikon, Tokyo, Japan) under bright-field illumination at 200× magnification.

### 4.4. Biochemical Analysis of Serum Indices

Uric acid (UA), creatinine (Cr), and blood urea nitrogen (BUN) levels in mouse serum were measured using a microplate reader at 450 nm, following the manufacturer’s protocols (Nanjing Jiancheng Technology Co., Ltd., Nanjing, China).

### 4.5. Elisa

Kidney tissues were homogenized and centrifuged to collect supernatants. Levels of IL-1β, IL-18, and TNF-α in serum and renal tissue supernatants were determined using ELISA kits (Wuhan GeneMei Biological Technology Co., Ltd., Wuhan, China) according to the manufacturer’s instructions.

### 4.6. 16S rRNA Sequencing Analysis

To characterize the taxonomic composition of the gut microbiota, fecal samples were collected one day before sacrifice. Thirty fecal samples passed sequencing and quality-control filtering and were included in the final microbiota analysis (*n* = 6 per group). Genomic DNA was extracted using the MagPure Soil DNA LQ Kit (Magen Biotech Co., Ltd., Guangdong, China) and sequenced on the Illumina NovaSeq 6000 system (Illumina, Inc., San Diego, CA, USA). Raw reads were processed with DADA2 within the QIIME2 framework (v2024.2.0) to generate an amplicon sequence variant (ASV) abundance table, and representative sequences were taxonomically annotated using the q2-feature-classifier. Alpha diversity was evaluated using Chao1, observed features, Shannon, Simpson, Pielou evenness, dominance, and Good’s coverage indices. Beta diversity was assessed using Bray–Curtis distances and visualized by principal coordinate analysis (PCoA) and non-metric multidimensional scaling (NMDS). Differences in overall microbial community structure were evaluated by permutational multivariate analysis of variance (PERMANOVA) and ANOSIM. Differentially abundant taxa were screened using non-parametric tests and linear discriminant analysis effect size (LEfSe).

### 4.7. Cell Culture and Treatments

Human renal tubular epithelial cells (HK-2, Cat. No. SNL-165) were obtained from SUNNCELL (Wuhan, China) and cultured in HK-2 complete medium supplemented with 10% fetal bovine serum (FBS) and 1% penicillin-streptomycin at 37 °C in a humidified 5% CO_2_ incubator. To assess cytotoxicity, cells were exposed to concentration gradients of cinnamaldehyde (0–200 μM), febuxostat (0–80 μM), or uric acid (0–1000 μg/mL). To evaluate the protective effect of cinnamaldehyde against uric acid-induced tubular epithelial injury, HK-2 cells were pre-treated with 400 μg/mL uric acid for 2 h, followed by co-incubation with low (6.25 μM) or high (25 μM) concentrations of cinnamaldehyde, or febuxostat (10 μM) as a positive control, for an additional 24 h.

### 4.8. Small-Interference-RNA (siRNA) Transfection

HK-2 cells were transfected with human AIF1- and CMPK2-specific small interfering RNAs (siRNAs) or a negative control siRNA (RiboBio Co., Ltd., Guangzhou, China) using riboFECT™ CP Reagent according to the manufacturer’s instructions. Cells were harvested 48 h post-transfection for subsequent analyses. The siRNA sequences are shown in Table 1.

### 4.9. Western Blotting

Total protein was extracted from cells and kidney tissues using RIPA lysis buffer supplemented with protease inhibitors. Equal amounts of protein were resolved on 10% SDS-PAGE gels (Affinibody, Cat. No. F10) and wet-transferred onto 0.45-μm PVDF membranes (Immobilon). Membranes were probed overnight at 4 °C with the indicated primary antibodies, washed extensively, and then incubated with HRP-conjugated secondary antibodies for 1 h at room temperature. Immunoreactive bands were visualized with enhanced chemiluminescence (ECL) substrate (Bio-Rad, Hercules, CA, USA), using GAPDH as an internal loading control. Chemiluminescent signals were captured on a Bio-Rad imaging system, and band intensities were quantified with ImageJ (1.x) software.

### 4.10. Immunofluorescence

For immunofluorescence staining, HK-2 cells were seeded onto glass coverslips in 6-well plates and treated as described in Section 2.7. Cells were fixed with 4% paraformaldehyde, blocked with 5% BSA for 30 min at room temperature, and then incubated overnight at 4 °C with primary antibodies against AIF1 (1:200), CMPK2 (1:200), and NLRP3 (1:200). After thorough washing with PBS, cells were incubated with the corresponding CY3- or FITC-conjugated secondary antibodies (1:100) for 50 min at room temperature in the dark. Nuclei were counterstained with DAPI for 10 min, and fluorescence images were acquired using a Nikon fluorescence microscope.

### 4.11. qPCR

Total RNA was isolated from HK-2 cells, and its concentration and purity were measured using a NanoDrop 2000 spectrophotometer (Thermo Fisher Scientific, Waltham, MA, USA). Complementary DNA (cDNA) was synthesized with an MMLV reverse transcriptase kit following the manufacturer’s protocol. Quantitative real-time PCR was subsequently performed on a Quantagene q225 system (KuBo Technology, Biejing, China) using the primers listed in Table 1. β-Actin served as the internal control, and relative gene expression was calculated by the 2^−ΔΔCT^ method.

### 4.12. Apoptosis and Mitochondrial Membrane Potential (ΔΨm) Assay

After treatment as described in Section 2.7, HK-2 cells were harvested. Apoptosis was assessed using an Annexin V-FITC/PI detection kit (Beyotime, Shanghai, China; Cat. No. C2006) according to the manufacturer’s protocol. Mitochondrial membrane potential (ΔΨm) was measured concurrently.

### 4.13. Transmission Electron Microscopy (TEM)

HK-2 cells were fixed with 2.5% glutaraldehyde, embedded, and cut into 80 nm ultrathin sections. The sections were double-stained with uranyl acetate and lead citrate, and mitochondrial ultrastructure was examined by transmission electron microscopy.

### 4.14. Statistical Analysis

Data are presented as mean +/– SD or mean +/– SEM, as indicated in the figure legends. Normality was assessed before parametric testing. For non-microbiota data, comparisons among multiple groups were analyzed by one-way ANOVA followed by Tukey’s multiple-comparison test; comparisons between two groups were analyzed using Student’s *t*-test when appropriate. For gut microbiota data, alpha-diversity indices were compared using non-parametric rank-based tests, beta diversity was evaluated using PERMANOVA and ANOSIM based on Bray–Curtis distances, and taxonomic differences among groups were explored using Wilcoxon or Kruskal–Wallis tests and LEfSe analysis. For taxa-level pairwise comparisons, both nominal *p* values and FDR-adjusted *p* values were considered when interpreting the results. Statistical analyses were performed using GraphPad Prism (11.0.2), QIIME2, and R (4.5.3) as appropriate. A value of *p* < 0.05 was considered statistically significant.

## 5. Conclusions

This study indicates that cinnamaldehyde alleviates hyperuricemia-associated renal injury in mice and reduces uric acid-induced injury in HK-2 cells. Its protective effect was associated with improved urate transporter expression, suppression of AIF1- and CMPK2/NLRP3-related inflammatory signaling, preservation of mitochondrial integrity. Additionally, exploratory 16S rRNA sequencing revealed compositional shifts in selected gut microbial taxa associated with cinnamaldehyde treatment; however, as beta-diversity differences did not reach statistical significance, these microbial observations should be interpreted as correlative and hypothesis-generating rather than as established mechanistic evidence. These findings support further investigation of cinnamaldehyde as a multi-target natural compound for hyperuricemia-related renal injury, while additional functional and causal studies are needed to confirm the key mechanisms.

## Figures and Tables

**Table 1:** The sequences of siRNA were used in this experiment.

siRNA	Sequence
SiRNA-AIF-1-1	AGACTCACCTAGAGCTAAA
SiRNA-AIF-1-2	AGAGATCTGCCATCCTAAA
SiRNA-AIF-1-3	CCTGACTTTCTCAGGATGA
SiRNA-CMPK2-1	GAACCAACTATCATTAGAA
SiRNA-CMPK2-2	TGCCAAATCTCCTGTGATT
SiRNA-CMPK2-3	GGCTGTCCTCTTAAAGTCA

## Data Availability

The original contributions presented in this study are included in the article/Supplementary Materials. Further inquiries can be directed to the corresponding author.

## Supplementary Materials

The following supporting information can be downloaded at: https://www.mdpi.com/article/10.3390/ph19081261/s1, Table S1: Summary of key gut microbiota statistics and interpretation.

## Abbreviations

The following abbreviations are used in this manuscript:

CA	Cinnamaldehyde
HUA	Hyperuricemia
AIF1	Allograft Inflammatory Factor 1
CMPK2	Cytidine/uridine Monophosphate Kinase 2
NLRP3	NOD-like receptor protein 3
HX	Hypoxanthine
PO	Potassium Oxonate
UA	Uric acid
HN	Hyperuricemic Nephropathy
Feb	Febuxostat
H&E	Hematoxylin and Eosin staining
HK2	Human Kidney-2
FBS	Fetal Bovine Serum
PBS	Fetal Bovine Serum
BUN	Blood urea nitrogen
CRE	Creatinine
ABCG2	ATP Binding Cassette Subfamily G Member 2
OAT1	Organic Anion Transporter 1
OCT2	Organic Cation Transporter 2
SLC2A9	Solute Carrier Family 2 Member 9
IL-18	Interleukin-18
IL-1β	Interleukin-1beta
TNF-α	Tumor Necrosis Factor-alpha
ASC	Apoptosis-associated speck-like protein containing a CARD
Caspase-1	Cysteine-aspartic acid protease 1
