# Supplementary material for: Cinnamaldehyde Attenuates Hyperuricemia-Associated Renal Injury by Modulating Urate Transporters and AIF1- and CMPK2/NLRP3-Related Inflammatory Signaling
Source: Pharmaceuticals (Basel). 2026 Aug 10;19(8):1261. doi: 10.3390/ph19081261 (PMC13516544; doi:10.3390/ph19081261)
Supplement: Supplementary file 1 [file pharmaceuticals-19-01261-s001.zip › pharmaceuticals-4428234-supplementary.pdf]

Table S1. Summary of key gut microbiota statistics and interpretation.

Analysis	Comparison or variable	Result	Interpretation
Sample size	Control, MOD, Feb, CA.L, CA.H	n = 6 per group	All 30 fecal samples passed sequencing/QC
Alpha diversity	Shannon, Chao1, observed features	Pairwise $P > 0.05$	No statistically significant diversity restoration
PERMANOVA	Control vs. MOD	$P = 0.267$	No significant global community separation
PERMANOVA	MOD vs. CA.L / MOD vs. CA.H	$P = 0.544$ / $P = 0.550$	No significant treatment-driven beta-diversity shift
ANOSIM	Control vs. MOD	$P = 0.326$	Consistent with non-significant beta-diversity separation
ANOSIM	MOD vs. CA.L / MOD vs. CA.H	$P = 0.423$ / $P = 0.404$	No significant treatment-driven community separation
Phylum ratio	Bacillota/Bacteroidota	Control 1.84; MOD 0.97; CA.H 0.91	The ratio was not restored by high-dose cinnamaldehyde
Selected genus	Escherichia-Shigella	Control 0.001%; MOD 4.30%; CA.H 0.004%	Directionally reduced by high-dose cinnamaldehyde; exploratory after FDR correction
